# Identification and Validation of Aging-Related Genes in Alzheimer’s Disease

**DOI:** 10.3389/fnins.2022.905722

**Published:** 2022-05-09

**Authors:** Qian Zhang, Jian Li, Ling Weng

**Affiliations:** ^1^Department of Neurosurgery, Xiangya Hospital, Central South University, Changsha, China; ^2^Hydrocephalus Center, Xiangya Hospital, Central South University, Changsha, China; ^3^Department of Neurology, Xiangya Hospital, Central South University, Changsha, China; ^4^National Clinical Research Center for Geriatric Disorders, Central South University, Changsha, China

**Keywords:** Alzheimer’s disease, aging, gene, WGCNA, bioinformatics

## Abstract

Aging is recognized as the key risk factor for Alzheimer’s disease (AD). This study aimed to identify and verify potential aging-related genes associated with AD using bioinformatics analysis. Aging-related differential expression genes (ARDEGs) were determined by the intersection of limma test, weighted correlation network analysis (WGCNA), and 1153 aging and senescence-associated genes. Potential biological functions and pathways of ARDEGs were determined by GO, KEGG, GSEA, and GSVA. Then, LASSO algorithm was used to identify the hub genes and the diagnostic ability of the five ARDEGs in discriminating AD from the healthy control samples. Further, the correlation between hub ARDEGs and clinical characteristics was explored. Finally, the expression level of the five ARDEGs was validated using other four GEO datasets and blood samples of patients with AD and healthy individuals. Five ARDEGs (GFAP, PDGFRB, PLOD1, MAP4K4, and NFKBIA) were obtained. For biological function analysis, aging, cellular senescence, and Ras protein signal transduction regulation were enriched. Diagnostic ability of the five ARDEGs in discriminating AD from the control samples demonstrated a favorable diagnostic value. Eventually, quantitative real-time reverse transcription-polymerase chain reaction (qRT-PCR) validation test revealed that compared with healthy controls, the mRNA expression level of PDGFRB, PLOD1, MAP4K4, and NFKBIA were elevated in AD patients. In conclusion, this study identified four ARDEGs (PDGFRB, PLOD1, MAP4K4, and NFKBIA) associated with AD. They provide an insight into potential novel biomarkers for diagnosing AD and monitoring progression.

## Introduction

Alzheimer’s disease (AD) is a neuro-degenerative disorder most prevalent in people over 65 years (Lashuel et al., 2002; Prince et al., 2013). It initially impacts memory and leads to progressive and irreversible cognitive decline and functional impairment, seriously affecting the patient quality of life (Barbe et al., 2018). As the population ages, the increasing incidence of AD imposes a heavy economic burden on family and society (Alzheimer’s Association, 2015; GBD 2016 Dementia Collaborators, 2019). Aging or senescence are recognized as the critical risk factors for AD (Saez-Atienzar and Masliah, 2020). Pathologically, senescent cells accumulate in aged tissues and have been shown to play causal roles in age-related diseases, including AD (Guerrero et al., 2021). Changes in the timing or nature of the cellular markers of normal senescence may be vital to understanding the events that convert normal senescence to neurodegeneration. Cellular senescence is a candidate mechanism that might be important for this conversion. Studies reported that during the development of AD, both post-mitotic cells [e.g., neurons (Wei et al., 2016)] and proliferating cells [e.g., astrocytes (Erol, 2010) and microglia (Tischer et al., 2016)] can produce a chronic state of cellular senescence. Therefore, abating senescent cells is considered a promising therapeutic approach to target the aging phenotype and thereby prevent or mitigate the onset and progression of AD (Childs et al., 2017; Saez-Atienzar and Masliah, 2020).

Gene expression analysis is becoming important in biological research (Fu et al., 2013). The availability of high-throughput transcriptomic sequencing data and clinical annotation in the Accelerating Medicines Partnership-Alzheimer’s Disease (AMP-AD) program allow us to study the altered transcriptional and associated biological pathways involved in AD (Meng and Mei, 2019). Several studies have utilized the gene expression datasets downloaded from the Gene Expression Omnibus (GEO) database to elucidate the biological mechanisms in the development of AD (Ma et al., 2019; Liu et al., 2020, 2021). Results from these bioinformatics analysis supply ponderable hints for understanding the pathogenesis molecular mechanisms of AD from a diverse perspective. However, no study focuses on which aging-related genes (ARGs) are critical for AD development using bioinformatics analysis. Therefore, in this study, we aimed to analyze the AD-related GEO datasets from the angle of the ARGs. The Aging-related DEGs (ARDEGs) were determined by the intersection of limma test, weighted correlation network analysis (WGCNA), and 1153 aging and senescence-associated genes. The potential biological functions and pathways of ARDEGs were determined by GO, KEGG, GSEA, and GSVA. Then, LASSO algorithms were used to identify the hub genes involved in AD development. In addition, the correlation between hub ARDEGs and clinical characteristics within AD we explored. Finally, the expression level of the four ARDEGs was validated using other four GEO datasets and clinical samples.

## Materials and Methods

### Downloading and Processing of Data

Microarray data, containing five transcription profiles [GSE132903 (Piras et al., 2019), GSE122063 (McKay et al., 2019), GSE5281 (Liang et al., 2007), GSE63060 (Sood et al., 2015), and GSE106241 (Marttinen et al., 2019)] were downloaded from the NCBI GEO database.^1^ The details of the datasets included in this analysis are listed in Table 1. GSE132903 dataset was acted as the training dataset. GSE122063, GSE63060, and GSE5281 datasets were used to validate the hub genes. We also utilized the GSE106241 (only included AD patients) to explore the relationships of hub genes with the clinical characteristics. Raw data or series matrix files were downloaded and processed, and the probe with maximal mean values of expression was annotated into the homologous gene symbol by means of the platform’s annotation information if multiple probes were matched with one gene.

**TABLE 1:** The details of the selected datasets.

Rank	Accession number	Platform	No. of samples	Sample source	Female:Male	Mean age of AD (year)	Mean age of ND (year)
1	GSE132903	GPL10558	97 AD and 98 ND	Middle temporal gyrus	96:99	85.02	84.98
2	GSE122063	GPL16699	56 AD and 44 ND	Frontal cortex, temporal cortex	68:32	78.6	80.9
3	GSE5281	GPL570	87 AD and 74 ND	Entorhinal cortex, hippocampus, medial temporal gyrus, posterior cingulate, superior frontal gyrus, and primary visual cortex	58:103	-	-
4	GSE106241	GPL24170	60 AD	Inferior temporal cortex	48:12	80.6	-
5	GSE63060	GPL6947	145 AD and 104 ND	Peripheral blood	161:88	75.4	72.38

*AD, Alzheimer’s disease; ND, non-demented controls.*

### Aging and Senescence-Associated Genes

A total of 1153 aging and senescence-associated genes were obtained from the Human Aging Genomic Resources^2^ and MSigDB gene sets^3^ [including GOBP regulation of cell aging (M16568), GOBP positive regulation of cell aging (M24705), and GOBP replicative senescence (M14683), Reactome cellular senescence (M27188), Biocarta longevity pathway (M13158), GOBP cell aging (M14701), Tang senescence Tp53 targets up (M11850), WP tca cycle in senescence (M40058), WP senescence and autophagy in cancer (M39619)] after deleting the duplicated genes.

### Analysis of Differential Expression Genes

After the data standardization and normalization of GSE132903 using the normalize Between Arrays function in the “limma” R package, principal component analysis (PCA) was conducted by using the “factoextra” R package. The DEGs between AD and non-demented controls (ND) were analyzed by using the “limma” R package. The DEGs were screened with the criteria of |Fold Change| > 0.58 and *p* < 0.05. A volcano plot and a heat map plot were depicted by using the R software ggplot2 package and “ComplexHeatmap” to show significantly deregulated genes, respectively.

### Enrichment Analysis

The “clusterProfiler” package was used to enrich the biological processes (BPs) of Gene Ontology (GO) and Kyoto Encyclopedia of Genes and Genomes (KEGG) pathways of DEGs. Gene set enrichment analysis (GSEA) was also conducted to identify the significantly differential functions between AD and ND. The gene sets of “c5.go.bp.v7.5.1.symbols” was downloaded from the MSigDB database. The gene set variation analysis (GSVA) algorithm was used to calculate the processes score using the “GSVA” R package.

### Identification of Co-expression Genes

Weighted Gene Co-expression Network Analysis (WGCNA) is an algorithm to cluster genes into different modules and uncover the relationships between modules and disease traits (Langfelder and Horvath, 2008). To comprehensively investigate the genetic mechanisms involved in the pathogenesis of AD, a co-expression network was constructed by the “WGCNA” package in R. The co-expression network was constructed only using the genes with the top 25% variance from the GSE132903 dataset. The dynamic cutting tree method was adopted to merge modules with the threshold of 0.25. Other criteria were used to construct the co-expression network: soft threshold power (β) based on the scale-free topology criterion (an independence index of *R*^2^ = 0.85) by using the pick Soft Threshold function; minimum genes of each module = 30. Pearson correlation analysis was adopted to uncover the potential correlations between modules and clinical characteristics of patients. The genes from the key modules were used for the GO and KEGG enrichment analysis.

### Identification of Aging-Related Differential Expression Genes

We intersected the DEGs, genes from key modules (WGCNA), and aging and senescence-associated genes to identify the ARDEGs. The overlapped genes were visualized with a heat-map. The biological processes and enrichment pathways were also discovered, as mentioned above.

### Identification of Hub Aging-Related Differential Expression Genes

We divided the GSE132903 dataset into the training (50%) and test (50%) cohorts. The least absolute shrinkage and selection operator (LASSO) logistic regression was used to identify the hub ARDEGs using the “glmnet” package. The minimum lambda was defined as the optimal value. The ARDEGs predicting score (ARGPS) was calculated in each sample. Receiver operating characteristic curve analysis was used to evaluate the diagnostic and discriminative value of the ARGPS in AD and ND. GSE122063, GSE63060, and GSE5281 datasets were used as the external validation datasets. A forest plot was used to visualize the odds ratio between AD and ND.

### Correlation Between Hub Aging-Related Differential Expression Genes and Clinical Characteristics in GSE106241

We enrolled another GSE106241 dataset which only had the AD patients, but with different clinical variables off setting the limitations of our discovery dataset. The relationships between genes and clinical traits (age, Braak stages, alpha-, beta-, gamma-secretase activity, and amyloid-beta 42 levels) were analyzed using the Spearman correlation analysis. Moreover, the expression level of hub genes in different Braak stages was tested using the Kruskal–Wallis test and depicted with a violin plot.

Construction of a TF-ARDEGs-miRNA network MiRNA and transcriptional factors (TF) usually participate in the process of gene transcription and post-transcription. Network analyst, a comprehensive network visual analytics platform for gene expression analysis, was used to predict the TF and miRNA of hub genes through the dataset collected from the RegNetwork repository. The interactional network of TF-ARDEGs-miRNA was further visualized in Cytoscape software.

### Patients With Alzheimer’s Disease and Healthy Controls

To further verify selected five hub ARDEGs, we recruited 15 patients with AD (case group) and 15 healthy controls (HC group, age, and sex-matched) from Xiangya hospital of Central South University. The selection criteria for AD and HC are the same as our published works (Jiao et al., 2021). Clinical characteristics of the samples are shown in Supplementary Table 1. The study protocol was approved by the Institutional Review Board of Xiangya Hospital of Central South University in China (Ethics number: 2019030501). Written informed consent was obtained from each participant or guardian.

### RNA Extraction and Quantitative Real-Time Reverse Transcription-Polymerase Chain Reaction

Peripheral blood monocytes (PBMCs) were isolated from the blood samples of AD and HC using Ficoll solution (Solarbio, Beijing, China). Total RNA was extracted from the PBMCs using TRIzol reagent (Takara, Kyoto, Japan) according to the manufacturer’s protocol. Then reverse transcription reactions were performed using 500 ng RNA and an Evo M-MLV reverse transcriptase kit (Accurate Biotechnology, Hunan, China) according to instructions. SYBR Green Pro Taq HS Kit (Accurate Biotechnology, Hunan, China) and 0.4 μmol of each primer pair were used to amplify the cDNA, then evaluated in an ABI 7500 real-time PCR system (Applied Bioscience, Foster City, CA, United States). The results were analyzed using the 2^–ΔΔ*Ct*^ method and expressed as the ratio of the internal control, ACTIN. The primer sequences used for the real-time PCR analysis are available in Table 2.

**TABLE 2:** Primer sequences for RT-qPCR.

Genes	Forward primer (5′-3′)	Reverse primer (5′-3′)
GFAP	AGGTCCATGTGGAGCTTGAC	GCCATTGCCTCATACTGCGT
PDGFRB	AGACACGGGAGAATACTTTTGC	AGTTCCTCGGCATCATTAGGG
PLOD1	AGACCAAGTATCCGGTGGTGT	CTTGAGCACGACCTCATCCAA
MAP4K4	GACTCCCCTGCAAAAAGTCTG	GTCCATAGGTGCCATTTCCAA
NFKBIA	CTCCGAGACTTTCGAGGAAATAC	GCCATTGTAGTTGGTAGCCTTCA

### Statistical Analysis

All statistical analyses were conducted using R software (version R-4.1.0). The Wilcoxon test was used for statistical analysis between two groups, and the Kruskal–Wallis test was selected flexibly when there were three or more groups. *P* < 0.05 was considered statistical significance. The significance level is denoted as follows: *P < 0.05, ^**^P < 0.01, ^***^P < 0.001, and ^****^P < 0.0001.

## Results

### Principal Component Analysis and Identification of Differential Expression Genes

**FIGURE 1:**
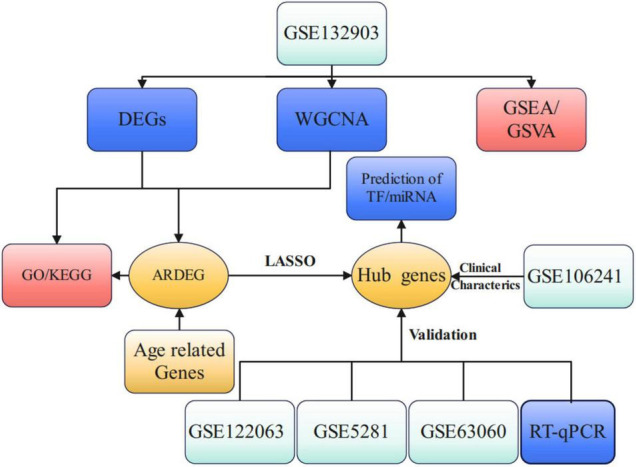
Study flow chart of present work. DEGs, differential expression genes; WGCNA, weighted gene co-expression network analysis; GSEA, gene set enrichment analysis; GSVA, gene set variation analysis; ARDEG, age-related differential expression genes; GO, gene ontology; KEGG, kyoto encyclopedia of genes and genomes; LASSO, least absolute shrinkage and selection operator; TF, transcriptional factors.

**FIGURE 2:**
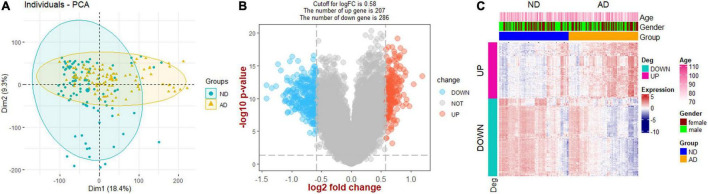
Screening of DEGs. **(A)** Principal component analysis (PCA). **(B)** Volcano plot showing the DEGs between Alzheimer’s disease and non-demented controls. **(C)** Heat map plot showing the expression patterns of DEGs. AD, Alzheimer’s disease; ND, non-demented controls.

The flow chart of this study is presented in Figure 1. PCA analysis showed that AD patients and ND controls were relatively discriminated into two groups (Figure 2A). A total of 493 DEGs were identified, including 207 upregulated genes and 286 downregulated genes (Figure 2B). Figure 2C indicated the expression patterns of the identified DEGs with a heat map.

### Analysis of Functional Enrichments

**FIGURE 3:**
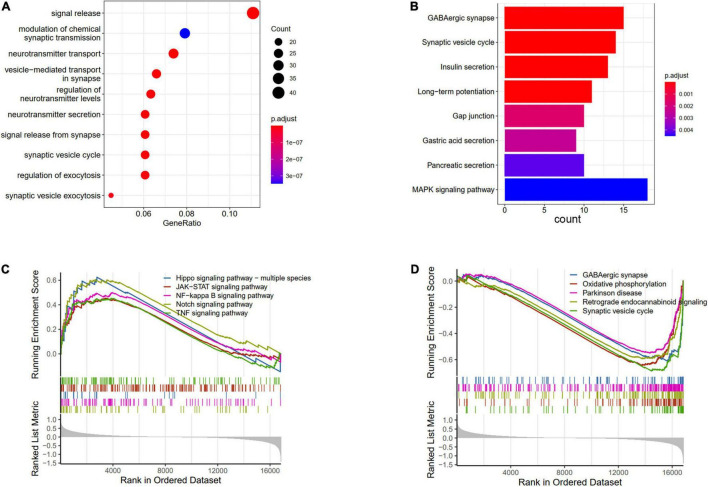
Enrichments analysis of DEGs between AD and ND. **(A)** GO biological processes of DEGs. **(B)** KEGG terms of DEGs enriched. GSEA results of upregulated **(C)** and downregulated **(D)** pathways.

The DEGs between AD and ND were used to conduct the enrichment analysis. The BP results showed that the signal release, modulation of chemical synaptic transmission, and neurotransmitter transport were enriched (Figure 3A). The pathways analysis showed that the GABAergic synapse, synaptic vesicle cycle, and MAPK signaling pathway were enriched in KEGG terms (Figure 3B). Furthermore, the GSVA results of BP indicated that regulation of gluconeogenesis, positive regulation of Notch receptor target, and pyroptosis processes were upregulated in AD patients, while nervous system process and neuropeptide signaling pathway were downregulated (Supplementary Figure 1). The GSEA results showed that compared with the ND controls, the Hippo signaling pathway, JAK-STAT signaling pathway, Notch signaling pathway, and TNF signaling pathway were increased in AD patients (Figure 3C), while GABAergic synapse, synaptic vesicle cycle, oxidative phosphorylation, and retrograde endocannabinoid signaling were decreased (Figure 3D).

### Co-expression Network Construction and Module Detection

**FIGURE 4:**
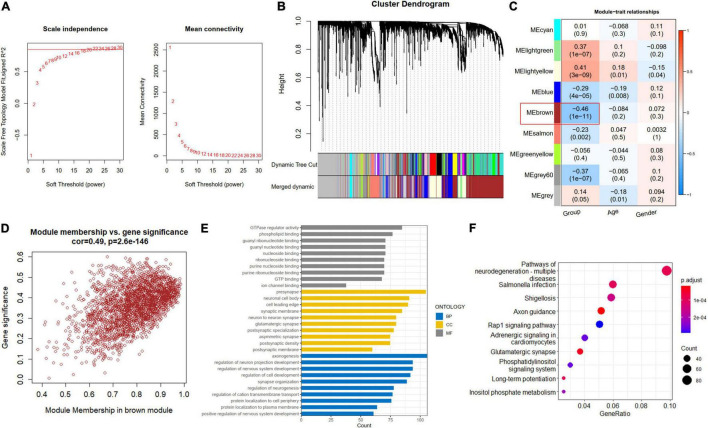
Construction of co-expression modules. **(A)** Scale-free fitting index analysis and mean connectivity of soft threshold power from 1 to 30. **(B)** Clustering dendrograms. According to dynamic tree cutting, the genes were clustered into different modules through hierarchical clustering with the threshold of 0.25. Each color represents each module. **(C)** Correlation heatmap between module eigengenes and clinical traits. **(D)** Scatterplot of genes in the brown module. **(E)** GO of biological processes (BP), cellular component (CC), and molecular functions (MF) for brown module. **(F)** KEGG brown module.

A soft-thresholding power of 22 was used to obtain approximate scale-free topology for the network (Figure 4A and Supplementary Figure 2). The top 25% variance genes (7215 genes) were clustered and merged into nine co-expression modules (Figure 4B). The correlation between module eigengene and clinical traits was explored by Pearson’s correlation analysis (Figure 4C). Results showed that the brown module (2419 genes) was significantly associated with the “Group” trait (i.e., AD and ND) and showed the strongest correlation (Figure 4D). Also, we performed function enrichment for the genes inside the brown module. The most enriched GO terms of the brown module included axonogenesis for BP, presynapse for cellular component (CC), and GTPase regulator activity for molecular function (MF) in Figure 4E. The KEGG terms were most enriched in the pathways of neurodegeneration in Figure 4F.

### Identification of a Least Absolute Shrinkage and Selection Operator Model With Aging-Related Genes

**FIGURE 5:**
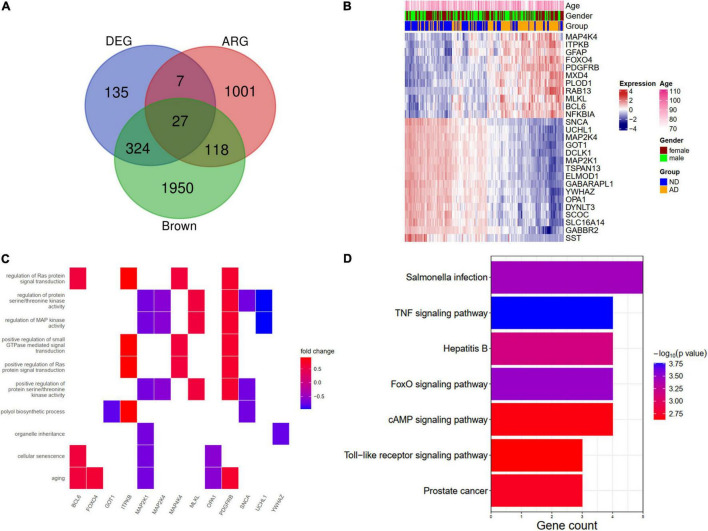
Identification and functional enrichment analysis of aging-related DEGs (ARDEGs). **(A)** Identification of ARDEGs with a venn diagram. **(B)** A heat map showing the expression of ARDEGs. **(C)** A heat map showing the biological processes and related ARDEGs. **(D)** KEGG pathways of ARDEGs.

We further overlapped the DEGs, the brown module genes (WGCNA), and aging-related genes. A total of 27 genes (ARDEGs) were intersected (Figure 5A). The heat map showed the expression patterns of these genes in AD patients and the controls (Figure 5B). An enrichment analysis of the 27 genes was also conducted. In BP results, the aging, cellular senescence, and regulation of Ras protein signal transduction were enriched, and the genes involved in these pathways were depicted in Figure 5C. The KEGG results indicated that salmonella infection and the TNF signaling pathway might participate in the pathogenesis of AD (Figure 5D).

**FIGURE 6:**
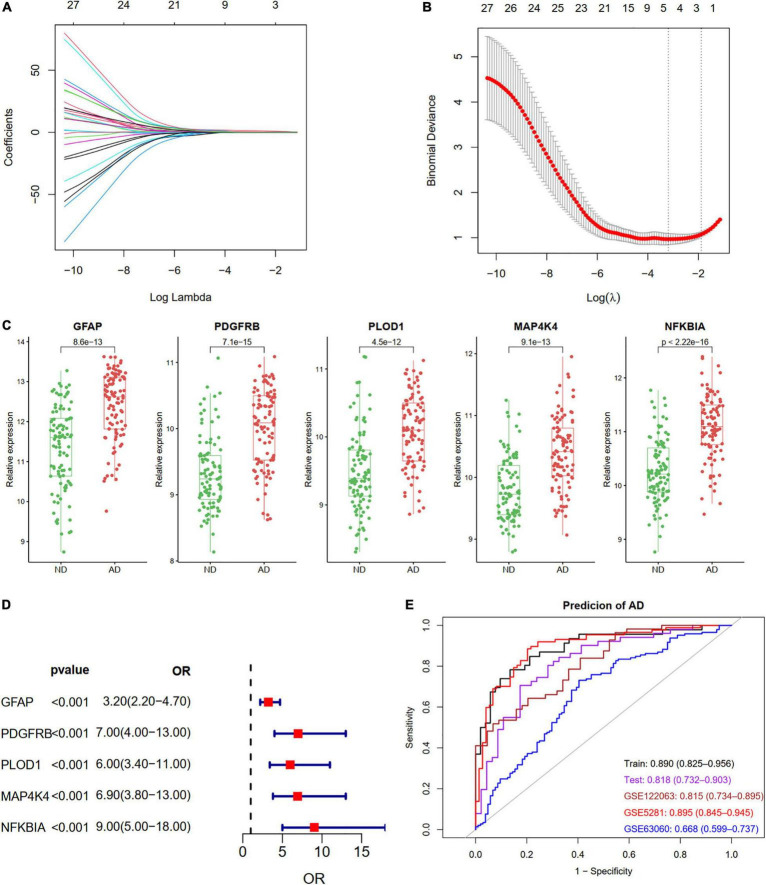
Construction a diagnostic model using the ARDEGs. **(A,B)** Feature selection by LASSO regression model in train dataset of GSE132903. **(A)** The coefficients change of different genes with different lambda. **(B)** By verifying the optimal parameter (lambda) in the LASSO model, the partial likelihood deviance (binomial deviance) curve was plotted vs. log (lambda). Dotted vertical lines were drawn based on 1 SE of the minimum criteria (the 1-SE criteria). Five features with non-zero coefficients were selected by optimal lambda. **(C)** Boxplot showing the relative expression of the five selected genes in GSE132903. **(D)** Forest plot of five genes based on univariate logistic regression. **(E)** ROC curves showing the five ARDEGs diagnostic ability in train and test dataset of GSE132903, GSE122063, GSE5281, and GSE63060.

We identified five ARDEGs (GFAP, PDGFRB, PLOD1, MAP4K4, and NFKBIA) with non-zero regression coefficients using the LASSO method and with the optimal lambda value of lambda.min = 0.0411 (Figures 6A,B). The coefficients and descriptions of the 27 ARDEGs are listed in Supplementary Table 2. The box plots and forest showed the significantly different levels of the five genes between AD and controls (Figures 6C,D). As shown in Figure 6E, the diagnostic ability of the five ARDEGs in discriminating AD from the control samples demonstrated a favorable diagnostic value, with an area under the curve (AUC) of 0.890 (95% CI 0.825–0.956) in the training set, an AUC of 0.818 (95% CI 0.732–0.903) in the test set, an AUC of 0.815 (95% CI 0.734–0.895) in the GSE122063 dataset, an AUC of 0.895 (95% CI 0.845–0.945) in the GSE5281 dataset, and an AUC of 0.668 (95% CI 0.599–0.737) in the GSE63060 dataset.

### Relationships of Hub Aging-Related Differential Expression Genes and Clinical Characteristics

**FIGURE 7:**
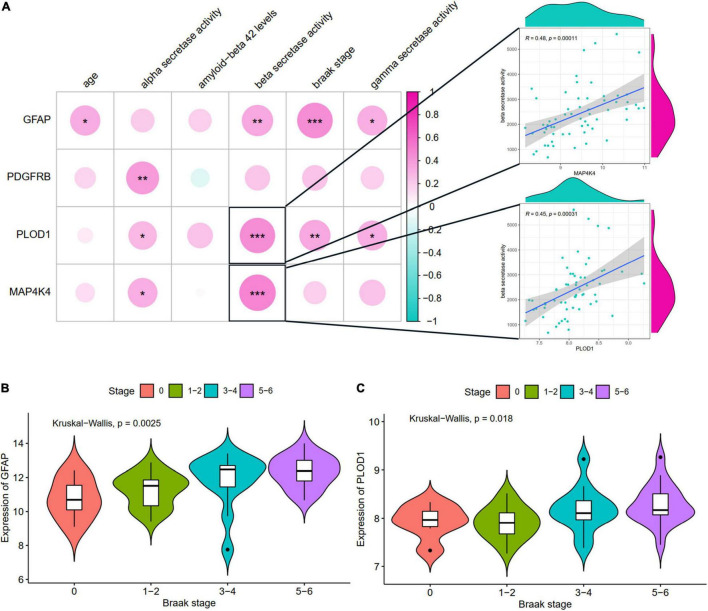
The correlation between hub ARDEGs and clinical characteristics in GSE106241. **(A)** The landscapes of the correlation between hub ARDEGs and clinical characteristics. The two respective scatterplots show hub ARDEGs and clinical characteristics with the highest correlation; **(B,C)** Shows the expression levels of GFAP and PLOD1 in different Braak stages, respectively. Significance level was denoted by *p-value < 0.05, **p-value < 0.01, ***p-value < 0.001.

The relationships of hub ARDEGs and clinical characteristics in GSE106241 (NFKBIA was not found in this dataset) were depicted in Figure 7 and Supplementary Figure 4. The relationship between age and the five ARDEGs (GFAP, PDGFRB, PLOD1, MAP4K4, and NFKBIA) was no significant difference (Supplementary Figure 3). With regards to clinical feature, four ARDEGs (GFAP, PDGFRB, PLOD1, MAP4K4) were almost positively related to more severity of clinical features (Figure 7A). Especially, PLOD1 and MAP4K4 were strongly correlated with beta-secretase activity. GFAP and PLOD1 were significantly associated with the Braak stages (Figures 7B,C).

### Prediction of miRNA and Transcriptional Factors of Hub Aging-Related Differential Expression Genes

A total of 130 miRNA and 124 TF were predicted for hub ARDEGs (Supplementary Table 3). The miRNA–ARDEGs interaction network is presented in Supplementary Figure 5A. The circular and squared nodes represent the ARDEGs and the miRNA, respectively. The size of a node depends on the degree of the node. For example, NFKBIA and MAP4K4 were interacted with 47 and 54 miRNA, while GFAP was only predicted 14 miRNA. The TF–ARDEGs interaction network is presented in Supplementary Figure 5B. The circular and diamond nodes represent the ARDEGs and the TF, respectively. GFAP, MAP4K4, NFKBIA, and PLOD1 were interacted with 7, 28, 65, and 56 TF, respectively. No TF was predicted for PDGFRB.

### Validation of Hub Aging-Related Differential Expression Genes by Other Gene Expression Omnibus Datasets and Quantitative Real-Time Reverse Transcription-Polymerase Chain Reaction in Clinical Samples

**FIGURE 8:**
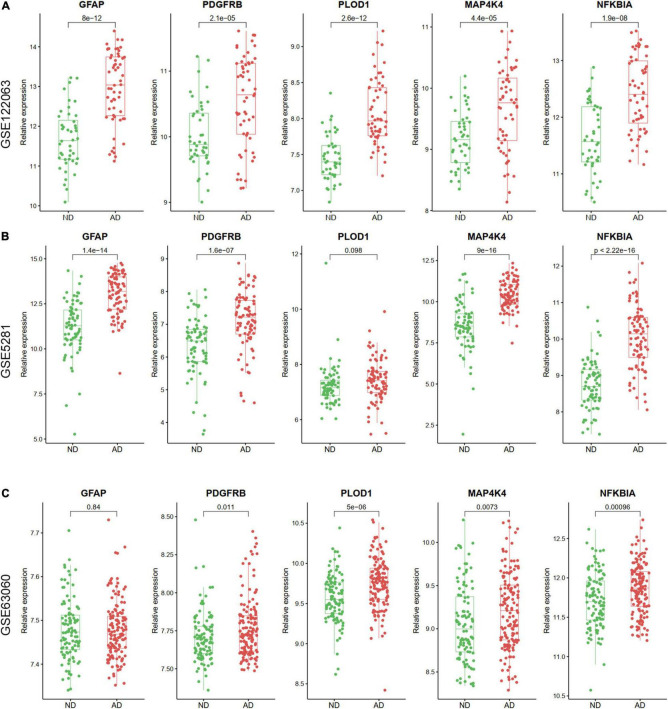
The relative expression levels of five hub ARDEGs in GSE122063 **(A)**, GSE5281 **(B)**, and GSE63060 **(C)** with box and scatter plots. Except for the expression of PLOD1 in GSE5281 and GFAP in GSE63060 did not show significant differences, the other genes were increased in AD samples.

The expression levels of the five ARDEGs (GFAP, PDGFRB, PLOD1, MAP4K4, and NFKBIA) were also verified in GSE122063, GSE5281, GSE63060 datasets. Except for the expression of PLOD1 in GSE5281, and GFAP in GSE63060 did not show significant differences, the other genes were increased in AD samples (Figure 8).

**FIGURE 9:**
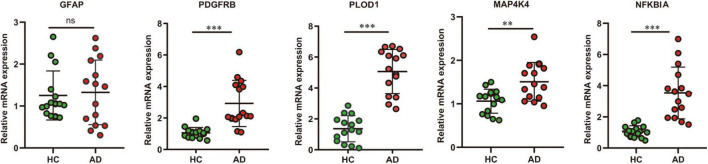
The result of quantitative real-time reverse transcription-polymerase chain reaction (qRT-PCR) illustrated the mRNA expression levels of GFAP, PDGFRB, PLOD1, MAP4K4, NFKBIA in patients with AD (*n* = 15) and healthy controls (*n* = 15). Significance level was denoted by ^**^p-value < 0.01, ^***^p-value < 0.001.

In addition, to verify the reliability of the five hubs ARDEGs, we collected blood samples from AD (*n* = 15) and healthy individuals (*n* = 15) to perform quantitative real-time reverse transcription-polymerase chain reaction (qRT-PCR). The results showed that compared with HCs, the mRNA expression levels of PDGFRB, PLOD1, MAP4K4, and NFKBIA were significantly elevated in AD patients (Figure 9), suggesting they may have the potential to be diagnostic biomarkers for AD.

## Discussion

Over 44 million people worldwide suffer from AD, and there is no effective treatment for AD due to an incomplete understanding of its etiology and pathogenesis (Robert et al., 2017). Cellular senescence, a state of permanent cell growth arrest, is believed to contribute importantly to aging and aging-related diseases (e.g., AD, cancer, pulmonary fibrosis). In addition, senescent astrocytes, microglia, endothelial cells, and neurons have been detected in the brain of AD patients and AD animal models (Liu, 2022). Nonetheless, although accumulated evidence highlights the role of cellular senescence in aging and AD, the mechanisms that promote cell senescence and how senescent cells contribute to AD neuro-pathophysiology remain largely unknown.

Recently, a few studies have highlighted the role of ARGs in different aging-related diseases. For example, Xiao et al. (2021) explored the potential roles of ARGs in glioma. Xu and Chen (2021) established a signature of ARGs for the prognosis of lung adenocarcinoma. Apart from cancers, the importance of ARGs in non-cancerous diseases also gained considerable attention. Such as, asthma (Yang et al., 2021), pulmonary fibrosis (He and Li, 2022), and chronic obstructive pulmonary disease (Du et al., 2019). However, bioinformatic analysis of ARGs in AD has not yet been performed, and crucial ARGs involved in the pathogenesis of AD are not confirmed. In the present study, we determined the ARDEGs through intersected DEGs (filtered by limma package), WGCNA, and 1153 ARGs (obtained from the Human Aging Genomic Resources and MSigDB gene sets). In addition, five hub ARDEGs (GFAP, PDGFRB, PLOD1, MAP4K4, and NFKBIA) were identified by LASSO logistic regression, and the diagnostic ability was validated using external datasets and clinical samples.

The biological function enrichment analysis showed the ARDEGs were enriched in aging, cellular senescence, and Ras protein signal transduction. Ras family GTPases (Ras, Rap1, and Rap2) and their downstream mitogen-activated protein kinases (ERK, JNK, and p38MAPK) and PI3K signaling cascades could modulate cellular senescence involved in the progression of AD (Stornetta and Zhu, 2011). For example, MAPK/ERK and PI3K/Akt pathway could be activated by beta-amyloid, enhancing vulnerability of neurons, exaggerating DNA damage in neurons, subsequently inducing neuron senescence (Tong et al., 2004; Kirouac et al., 2017). In patient, increased expression of Ras protein positively correlated with Aβ levels in the brain of patients with AD. What’s more, in animal, decyltriphenylphosphonium, a selective Ras protein inhibitor, could alleviate AD progression by suppressing MEK1/2-ERK1/2 activity (Muraleva et al., 2021). These works illustrate the Ras family protein plays an active role in AD. However, further works are needed to investigate the crosstalk between these five ARDEGs and the Ras signal transduction protein in AD.

Glial Fibrillary Acidic Protein (GFAP), one of the major intermediate filament proteins of mature astrocytes, is used as a marker to distinguish astrocytes from other glial cells during development (Eng et al., 2000). As long ago as 2005, studies explored the importance of GFAP in AD. Maruyama et al. (2005) found the higher expression of GFAP in the AD patients’ hippocampus. In the same year, another study showed caloric restriction in mice could decrease the expression of GFAP (astrocytic activation), thus retard the progression of AD, suggesting the potential therapy of GFAP for AD (Patel et al., 2005). Similarly, GFAP has been demonstrated to accumulate in a variety of neuro-degenerative diseases, such as Huntington disease (Rosenberg et al., 1981) and multiple sclerosis (Petzold, 2015). Seemingly, the accumulation of GFAP in neuro-degenerative diseases has been attributed to secondary astrocytic gliosis, which is an important shared pathologic process in neuro-degenerative diseases. However, Clairembault et al. (2014) showed patients with Parkinson’s disease, but not progressive supranuclear palsy and multiple system atrophy, had significant higher GFAP expression levels in their colonic biopsies. The result hints the potential diagnostic capability of GFAP for discriminating different neuro-degenerative diseases. More importantly, with the advancement of sensitivity of test technology, a recent study detected that plasma GFAP is higher in preclinical AD patients than in normal cognitive individuals (Oeckl et al., 2019). And also detected higher in symptomatic stages of AD than in patients with mild cognitive impairment, which suggests that plasma GFAP is a sensitive biomarker for diagnosing the early stages of AD (Benedet et al., 2021). In this study, we found elevated transcription expression of GFAP besides in GSE63060. A possible explanation for this discrepancy may be the different sample sources and diverse composition of study participants. The samples in GSE63060 obtained from peripheral blood, and the samples in other datasets obtained from brain tissue. We also validated the results using clinical blood samples, and found no significant difference of mRNA level of GFAP between AD and healthy control. It corroborates the results of our bioinformatics analysis. However, the relatively smaller sample size may lead to inherent bias. Further work will need to validate the expression level of GFAP from the protein level with larger sample size.

Platelet-Derived Growth Factor Receptor Beta (PDGFRB) is a dimeric receptor tyrosine kinase that plays critical roles in cell growth, survival, and differentiation (Gronwald et al., 1988). In addition, PDGFRB is a marker of pericyte, which controlling key neurovascular functions during brain aging (Bell et al., 2010). Numerous studies investigated the role of PDGFRB in the development of AD. For example, the decreased expression of PDGFRB in the precuneus is associated with fibrinogen leakage, reduced oxygenation, and related to fibrillar amyloid-β accumulation (Miners et al., 2018). The elevated soluble PDGFRB in cerebrospinal fluid correlates with the severity of blood-brain barrier breakdown and pericyte injury, an important pathologic process in AD (Sagare et al., 2015). In animal studies, Bell et al. (2010) reported that mice lacking PDGFRB showed age-dependent vascular damage and preceded degenerative neuronal changes, learning and memory deficits, and neuroinflammatory responses. Also, Kisler et al. (2017) found PDGFRB knockout mice presented with a neurovascular uncoupling, reduced oxygen supply to the brain, and over metabolic stress. Besides AD, studies reported the links between PDGFRB and other neuro-degenerative diseases. For example, primary familial brain calcification has recently been linked to heterozygous mutations in PDGFRB gene (Westenberger et al., 2019). And mutation of the PDGFRB gene is a cause of idiopathic basal ganglia calcification (Nicolas et al., 2013a,b). In the present study, PDGFRB in PBMCs increased in AD compared with healthy controls, the same as our bioinformatics analysis. Thus, whether soluble PDGFRB in serum could be a biomarker for monitoring the severity of AD could be considered in the future works.

Procollagen-Lysine, 2-Oxoglutarate 5-Dioxygenase 1 (PLOD1), also named as lysyl hydroxylase. It is essential for hydroxylation of lysine residues in collagen telopeptides and collagen cross-link formation (Tan et al., 2019). Chong et al. (2013) found that PLOD1, KIR2DL5A, SLC2A8 significantly upregulated in the peripheral blood leukocytes in fast progressed AD, suggesting its potential role as prognostic blood biomarkers to predict progression in early AD. Contrary to Chong et al. (2013), Gao et al. (2019) reported that serum concentration and expression levels of POLD1 in lymphocytes were reduced in AD patients. In animal study, POLD1 knockdown led to premature senescence and increased DNA damage in primary neuronal cells of senescence-accelerated mouse prone 8 mice, suggesting that the deficiency of POLD1 may aggravate AD progression. In our study, we found mRNA level of PLOD1 was elevated in PBMCs in AD. Previous studies showed AD could affect peripheral blood mononuclear cells (PBMCs), and PBMCs could aid the diagnosis of different forms of dementia and monitor disease progression (Arosio et al., 2014). Further work needs to investigate the mechanism of whether PLOD1 affect the progression of AD and whether PLOD1 alone in serum or PBMCs could be used to detect the different stage of AD using a large cohort.

Mitogen-Activated Protein Kinase Kinase Kinase Kinase 4 (MAP4K4) is a member of the serine/threonine-protein kinase family, which play a role in response to environmental stress and cytokines (Yao et al., 1999). Studies explored the relationship between MAP4K4 and neuro-degenerative diseases. For example, Wu et al. (2019) reported MAP4K4 promoted motor neurons death in amyotrophic lateral sclerosis, suppressing the expression of MAP4K4 not only promoted survival but prevented neurite degeneration in motor neurons. In addition, Bos et al. (2019) identified a small molecular drug (URMC-099), targeting the MAP4K family, functioned as a neuroprotective role *via* inhibiting endoplasmic reticulum stress in human motor neurons. Also, militarinone alkaloids, a potent neuro-protective molecule, enhanced the neurite outgrowth through MAP4K4 (Schroder et al., 2015). Limited studies talked about the relationship between MAP4K4 and AD. Jian et al. (2021) found MAP4K4 in glial cells could influence the occurrence and development of AD through ligand-receptor axis communication. Considering the protective role of inhibiting MAP4K4 in neurons, future research needs to investigate whether MAP4K4 could mediates the progression of AD in animal studies.

As for NFKB Inhibitor Alpha (NFKBIA), this protein interacts with REL dimers to inhibit NF-κB/REL complexes, which involved in immune and inflammatory responses (Haskill et al., 1991). Already back in 1999, Kaltschmidt et al. (1999) described the decreased immunoreactivity of NF-κB around various plaque stages of AD patients, and NF-κB activity protects neurons against oxidative stress (Heck et al., 1999). However, pharmacological intervention targeting microglial NF-κB could retard the progression of Parkinson’s disease through inhibiting the neuroinflammation (Wilms et al., 2003). Recently, Ryu et al. (2021) reported NF-κB signaling pathways are enriched in longevity-related genes, elevated NFKBIA inhibit the expression of NF-κB signaling pathways, leading to cognitive decline, dementia, and AD. Hence, inhibiting the expression of NFKBIA could be a target for the treatment of AD.

Finally, as future interventions targeting these hub ARDEGs and provide potential mechanism research, we established a miRNA-gene, and TF-gene network and found that MAP4K4 and NFKB1A was predicted to have the greatest miRNA and TF abundance, which provide clues about the molecular links between these ARDEGs and AD.

Several limitations existed in our study:

1. We did not obtain the brain tissue from patients with AD, and the expression of these ARDEGs was only validated using blood samples.
2. Although we validated our results using other datasets and clinical samples, our interpretation may contain some bias because relatively smaller samples and data came from mRNA expression. In future work, we intend to conduct an ELISA experiment (from protein level) for validating the diagnostic role of these five ARDEGs in larger samples.
3. We have not conducted an *in vitro* and *in vivo* experiment to verify the mechanisms underlying these ARDEGs in AD.

## Conclusion

In conclusion, we identified and validated a signature of four aging-related genes (PDGFRB, PLOD1, MAP4K4, and NFKBIA) associated with AD. These genes may participate in the initiation and progression of AD, and they provide an insight into potential novel biomarkers for diagnosing AD and monitoring progression.

## Data Availability Statement

Publicly available datasets were analyzed in this study. This data can be found here: GSE132903, GSE122063, GSE5281, GSE63060, and GSE106241 were downloaded from the NCBI GEO database (https://www.ncbi.nlm.nih.gov/geo/). Aging and Senescence-Associated Genes were obtained from the Human Aging Genomic Resources (https://genomics.senescence.info/) and MSigDB gene sets (http://www.gsea-msigdb.org/gsea/msigdb/index.jsp).

## Ethics Statement

The studies involving human participants were reviewed and approved by Institutional Review Board of Xiangya Hospital of Central South University in China (Ethics number: 2019030501). The patients/participants provided their written informed consent to participate in this study.

## Author Contributions

QZ and LW conceived and designed the study, conducted bioinformatic analyses, and wrote the manuscript. JL and LW provided insightful guidance and feedback for the study. All authors contributed to the article, read, and approved the final version of the manuscript.

## Conflict of Interest

The authors declare that the research was conducted in the absence of any commercial or financial relationships that could be construed as a potential conflict of interest.

## Publisher’s Note

All claims expressed in this article are solely those of the authors and do not necessarily represent those of their affiliated organizations, or those of the publisher, the editors and the reviewers. Any product that may be evaluated in this article, or claim that may be made by its manufacturer, is not guaranteed or endorsed by the publisher.

## Footnotes

1
https://www.ncbi.nlm.nih.gov/geo/

2
https://genomics.senescence.info/

3
http://www.gsea-msigdb.org/gsea/msigdb/index.jsp

## Supplementary Material

Supplementary Figure 1 Gene set variation analysis showed the 20 up and down regulated biological processes in AD patients. Regulation of gluconeogenesis, positive regulation of Notch receptor target, and pyroptosis processes were upregulated in AD, while nervous system process and neuropeptide signaling pathway were downregulated.

Supplementary Figure 2 Checking the scale-free topology using soft-thresholds with β = 22.

Supplementary Figure 3 Scatterplots showed no significant relationship between the five hub ARDEGs and age. Significance level was denoted by **p*-value < 0.05, ^**^*p*-value < 0.01, ^***^*p*-value < 0.001.

Supplementary Figure 4 **(A,B)** Showed the expression levels of MAP4K4 and PDGFRB in different Braak stages, respectively. **(C–H)** Scatterplots show the significant relationship between hub ARDEGs and clinical characteristics. Significance level was denoted by **p*-value < 0.05, ^**^*p*-value < 0.01, ^***^*p*-value < 0.001.

Supplementary Figure 5 The Predicted microRNA **(A)** and transcription factors **(B)** for the selected five hub ARDEGs. The circular nodes represent the ARDEGs, and the square nodes represent miRNA **(A)** or transcription factors **(B)**, respectively. The size of a node depends on the degree of the node.

## References

[B1] Alzheimer’s Association. (2015). 2015 Alzheimer’s disease facts and figures. *Alzheimers Dement.* 11 332–384. 10.1016/j.jalz.2015.02.003 25984581

[B2] Arosio B. D’Addario C. Gussago C. Casati M. Tedone E. Ferri E. (2014). Peripheral blood mononuclear cells as a laboratory to study dementia in the elderly. *Biomed. Res. Int.* 2014:169203. 10.1155/2014/169203 24877062 PMC4022117

[B3] Barbe C. Jolly D. Morrone I. Wolak-Thierry A. Drame M. Novella J. L. (2018). Factors associated with quality of life in patients with Alzheimer’s disease. *BMC Geriatr.* 18:159. 10.1186/s12877-018-0855-7 29986669 PMC6038200

[B4] Bell R. D. Winkler E. A. Sagare A. P. Singh I. LaRue B. Deane R. (2010). Pericytes control key neurovascular functions and neuronal phenotype in the adult brain and during brain aging. *Neuron.* 68 409–427. 10.1016/j.neuron.2010.09.043 21040844 PMC3056408

[B5] Benedet A. L. Mila-Aloma M. Vrillon A. Ashton N. J. Pascoal T. A. Lussier F. (2021). Differences between plasma and cerebrospinal fluid glial fibrillary acidic protein levels across the alzheimer disease continuum. *JAMA Neurol.* 78 1471–1483. 10.1001/jamaneurol.2021.3671 34661615 PMC8524356

[B6] Bos P. H. Lowry E. R. Costa J. Thams S. Garcia-Diaz A. Zask A. (2019). Development of MAP4 kinase inhibitors as motor neuron-protecting agents. *Cell Chem. Biol.* 26 1703–1715.e37. 10.1016/j.chembiol.2019.10.005 31676236 PMC7253076

[B7] Childs B. G. Gluscevic M. Baker D. J. Laberge R. M. Marquess D. Dananberg J. (2017). Senescent cells: an emerging target for diseases of ageing. *Nat. Rev. Drug Discov.* 16 718–735. 10.1038/nrd.2017.116 28729727 PMC5942225

[B8] Chong M. S. Goh L. K. Lim W. S. Chan M. Tay L. Chen G. (2013). Gene expression profiling of peripheral blood leukocytes shows consistent longitudinal downregulation of TOMM40 and upregulation of KIR2DL5A, PLOD1, and SLC2A8 among fast progressors in early Alzheimer’s disease. *J. Alzheimers Dis.* 34 399–405. 10.3233/JAD-121621 23234877

[B9] Clairembault T. Kamphuis W. Leclair-Visonneau L. Rolli-Derkinderen M. Coron E. Neunlist M. (2014). Enteric GFAP expression and phosphorylation in Parkinson’s disease. *J. Neurochem.* 130 805–815. 10.1111/jnc.12742 24749759

[B10] Du X. Yuan L. Wu M. Men M. He R. Wang L. (2019). Variable DNA methylation of aging-related genes is associated with male COPD. *Respir. Res.* 20:243. 10.1186/s12931-019-1215-7 31684967 PMC6829949

[B11] Eng L. F. Ghirnikar R. S. Lee Y. L. (2000). Glial fibrillary acidic protein: GFAP-thirty-one years (1969-2000). *Neurochem. Res.* 25 1439–1451. 10.1023/a:1007677003387 11059815

[B12] Erol A. (2010). Are paradoxical cell cycle activities in neurons and glia related to the metabolic theory of Alzheimer’s disease? *J. Alzheimers Dis.* 19 129–135. 10.3233/JAD-2010-1211 20061632

[B13] Fu W. Xie W. Zhang Z. Wang S. Wu Q. Liu Y. (2013). Exploring valid reference genes for quantitative real-time PCR analysis in *Plutella xylostella* (Lepidoptera: Plutellidae). *Int. J. Biol. Sci.* 9 792–802. 10.7150/ijbs.5862 23983612 PMC3753443

[B14] Gao S. Zhang X. Song Q. Liu J. Ji X. Wang P. (2019). POLD1 deficiency is involved in cognitive function impairment in AD patients and SAMP8 mice. *Biomed. Pharmacother.* 114:108833. 10.1016/j.biopha.2019.108833 30978525

[B15] GBD 2016 Dementia Collaborators (2019). Global, regional, and national burden of Alzheimer’s disease and other dementias, 1990-2016: a systematic analysis for the global burden of disease study 2016. *Lancet Neurol.* 18 88–106. 10.1016/S1474-4422(18)30403-4 30497964 PMC6291454

[B16] Gronwald R. G. Grant F. J. Haldeman B. A. Hart C. E. O’Hara P. J. Hagen F. S. (1988). Cloning and expression of a cDNA coding for the human platelet-derived growth factor receptor: evidence for more than one receptor class. *Proc. Natl. Acad. Sci. U.S.A.* 85 3435–3439. 10.1073/pnas.85.10.3435 2835772 PMC280226

[B17] Guerrero A. De Strooper B. Arancibia-Carcamo I. L. (2021). Cellular senescence at the crossroads of inflammation and Alzheimer’s disease. *Trends Neurosci.* 44 714–727. 10.1016/j.tins.2021.06.007 34366147

[B18] Haskill S. Beg A. A. Tompkins S. M. Morris J. S. Yurochko A. D. Sampson-Johannes A. (1991). Characterization of an immediate-early gene induced in adherent monocytes that encodes I kappa B-like activity. *Cell* 65 1281–1289. 10.1016/0092-8674(91)90022-q 1829648

[B19] He J. Li X. (2022). Identification and validation of aging-related genes in idiopathic pulmonary fibrosis. *Front. Genet.* 13:780010. 10.3389/fgene.2022.780010 35211155 PMC8863089

[B20] Heck S. Lezoualc’h F. Engert S. Behl C. (1999). Insulin-like growth factor-1-mediated neuroprotection against oxidative stress is associated with activation of nuclear factor kappaB. *J. Biol. Chem.* 274 9828–9835. 10.1074/jbc.274.14.9828 10092673

[B21] Jian C. Wei L. Mo R. Li R. Liang L. Chen L. (2021). Microglia mediate the occurrence and development of Alzheimer’s disease through ligand-receptor axis communication. *Front. Aging Neurosci.* 13:731180. 10.3389/fnagi.2021.731180 34616287 PMC8488208

[B22] Jiao B. Liu H. Guo L. Liao X. Zhou Y. Weng L. (2021). Performance of plasma amyloid beta, total tau, and neurofilament light chain in the identification of probable Alzheimer’s disease in South China. *Front. Aging Neurosci.* 13:749649. 10.3389/fnagi.2021.749649 34776933 PMC8579066

[B23] Kaltschmidt B. Uherek M. Wellmann H. Volk B. Kaltschmidt C. (1999). Inhibition of NF-kappaB potentiates amyloid beta-mediated neuronal apoptosis. *Proc. Natl. Acad. Sci. U.S.A.* 96 9409–9414. 10.1073/pnas.96.16.9409 10430956 PMC17796

[B24] Kirouac L. Rajic A. J. Cribbs D. H. Padmanabhan J. (2017). Activation of Ras-ERK signaling and GSK-3 by amyloid precursor protein and amyloid beta facilitates neurodegeneration in Alzheimer’s disease. *eNeuro* 4:ENEURO.0149-16.2017. 10.1523/ENEURO.0149-16.2017 28374012 PMC5367084

[B25] Kisler K. Nelson A. R. Rege S. V. Ramanathan A. Wang Y. Ahuja A. (2017). Pericyte degeneration leads to neurovascular uncoupling and limits oxygen supply to brain. *Nat. Neurosci.* 20 406–416. 10.1038/nn.4489 28135240 PMC5323291

[B26] Langfelder P. Horvath S. (2008). WGCNA: an R package for weighted correlation network analysis. *BMC Bioinform.* 9:559. 10.1186/1471-2105-9-559 19114008 PMC2631488

[B27] Lashuel H. A. Hartley D. Petre B. M. Walz T. Lansbury P. T. Jr. (2002). Neurodegenerative disease: amyloid pores from pathogenic mutations. *Nature* 418:291. 10.1038/418291a 12124613

[B28] Liang W. S. Dunckley T. Beach T. G. Grover A. Mastroeni D. Walker D. G. (2007). Gene expression profiles in anatomically and functionally distinct regions of the normal aged human brain. *Physiol. Genomics* 28 311–322. 10.1152/physiolgenomics.00208.2006 17077275 PMC2259385

[B29] Liu L. Wu Q. Zhong W. Chen Y. Zhang W. Ren H. (2020). Microarray analysis of differential gene expression in Alzheimer’s disease identifies potential biomarkers with diagnostic value. *Med. Sci. Monit.* 26:e919249. 10.12659/MSM.919249 31984950 PMC7001516

[B30] Liu R. M. (2022). Aging, cellular senescence, and Alzheimer’s disease. *Int. J. Mol. Sci.* 23:1989. 10.3390/ijms23041989 35216123 PMC8874507

[B31] Liu Z. Li H. Pan S. (2021). Discovery and validation of key biomarkers based on immune infiltrates in Alzheimer’s disease. *Front. Genet.* 12:658323. 10.3389/fgene.2021.658323 34276768 PMC8281057

[B32] Ma G. Liu M. Du K. Zhong X. Gong S. Jiao L. (2019). Differential expression of mRNAs in the brain tissues of patients with Alzheimer’s disease based on GEO expression profile and its clinical significance. *Biomed. Res. Int.* 2019:8179145. 10.1155/2019/8179145 30918899 PMC6413412

[B33] Marttinen M. Paananen J. Neme A. Mitra V. Takalo M. Natunen T. (2019). A multiomic approach to characterize the temporal sequence in Alzheimer’s disease-related pathology. *Neurobiol. Dis.* 124 454–468. 10.1016/j.nbd.2018.12.009 30557660

[B34] Maruyama N. Ishigami A. Kondo Y. (2005). [Molecular abnormality in aging: its contribution to clinical pathology]. *Rinsho Byori.* 53 728–734. 16190359

[B35] McKay E. C. Beck J. S. Khoo S. K. Dykema K. J. Cottingham S. L. Winn M. E. (2019). Peri-infarct upregulation of the oxytocin receptor in vascular dementia. *J. Neuropathol. Exp. Neurol.* 78 436–452. 10.1093/jnen/nlz023 30990880 PMC6467199

[B36] Meng G. Mei H. (2019). Transcriptional dysregulation study reveals a core network involving the progression of Alzheimer’s disease. *Front. Aging Neurosci.* 11:101. 10.3389/fnagi.2019.00101 31133844 PMC6513962

[B37] Miners J. S. Schulz I. Love S. (2018). Differing associations between Abeta accumulation, hypoperfusion, blood-brain barrier dysfunction and loss of PDGFRB pericyte marker in the precuneus and parietal white matter in Alzheimer’s disease. *J. Cereb. Blood Flow Metab.* 38 103–115. 10.1177/0271678X17690761 28151041 PMC5757436

[B38] Muraleva N. A. Kolosova N. G. Stefanova N. A. (2021). MEK1/2-ERK pathway alterations as a therapeutic target in sporadic Alzheimer’s disease: a study in senescence-accelerated OXYS rats. *Antioxidants (Basel)* 10:1058. 10.3390/antiox10071058 34208998 PMC8300733

[B39] Nicolas G. Pottier C. Charbonnier C. Guyant-Marechal L. Le Ber I. Pariente J. (2013a). Phenotypic spectrum of probable and genetically-confirmed idiopathic basal ganglia calcification. *Brain* 136(Pt 11) 3395–3407. 10.1093/brain/awt255 24065723

[B40] Nicolas G. Pottier C. Maltete D. Coutant S. Rovelet-Lecrux A. Legallic S. (2013b). Mutation of the PDGFRB gene as a cause of idiopathic basal ganglia calcification. *Neurology* 80 181–187. 10.1212/WNL.0b013e31827ccf34 23255827

[B41] Oeckl P. Halbgebauer S. Anderl-Straub S. Steinacker P. Huss A. M. Neugebauer H. (2019). Glial fibrillary acidic protein in serum is increased in Alzheimer’s disease and correlates with cognitive impairment. *J. Alzheimers Dis.* 67 481–488. 10.3233/JAD-180325 30594925

[B42] Patel N. V. Gordon M. N. Connor K. E. Good R. A. Engelman R. W. Mason J. (2005). Caloric restriction attenuates abeta-deposition in Alzheimer transgenic models. *Neurobiol. Aging* 26 995–1000. 10.1016/j.neurobiolaging.2004.09.014 15748777

[B43] Petzold A. (2015). Glial fibrillary acidic protein is a body fluid biomarker for glial pathology in human disease. *Brain Res.* 1600 17–31. 10.1016/j.brainres.2014.12.027 25543069

[B44] Piras I. S. Krate J. Delvaux E. Nolz J. Mastroeni D. F. Persico A. M. (2019). Transcriptome changes in the Alzheimer’s disease middle temporal gyrus: importance of RNA metabolism and mitochondria-associated membrane genes. *J. Alzheimers Dis.* 70 691–713. 10.3233/JAD-181113 31256118

[B45] Prince M. Bryce R. Albanese E. Wimo A. Ribeiro W. Ferri C. P. (2013). The global prevalence of dementia: a systematic review and metaanalysis. *Alzheimers Dement.* 9 63–75.e2. 10.1016/j.jalz.2012.11.007 23305823

[B46] Robert J. Button E. B. Stukas S. Boyce G. K. Gibbs E. Cowan C. M. (2017). High-density lipoproteins suppress Abeta-induced PBMC adhesion to human endothelial cells in bioengineered vessels and in monoculture. *Mol. Neurodegener.* 12:60. 10.1186/s13024-017-0201-0 28830501 PMC5568306

[B47] Rosenberg R. N. Ivy N. Kirkpatrick J. Bay C. Nyhan W. L. Baskin F. (1981). Joseph disease and Huntington disease: protein patterns in fibroblasts and brain. *Neurology* 31 1003–1014. 10.1212/wnl.31.8.1003 6455606

[B48] Ryu S. Han J. Norden-Krichmar T. M. Zhang Q. Lee S. Zhang Z. (2021). Genetic signature of human longevity in PKC and NF-kappaB signaling. *Aging Cell* 20:e13362. 10.1111/acel.13362 34197020 PMC8282271

[B49] Saez-Atienzar S. Masliah E. (2020). Cellular senescence and Alzheimer disease: the egg and the chicken scenario. *Nat. Rev. Neurosci.* 21 433–444. 10.1038/s41583-020-0325-z 32601397 PMC12548380

[B50] Sagare A. P. Sweeney M. D. Makshanoff J. Zlokovic B. V. (2015). Shedding of soluble platelet-derived growth factor receptor-beta from human brain pericytes. *Neurosci. Lett.* 607 97–101. 10.1016/j.neulet.2015.09.025 26407747 PMC4631673

[B51] Schroder P. Forster T. Kleine S. Becker C. Richters A. Ziegler S. (2015). Neuritogenic militarinone-inspired 4-hydroxypyridones target the stress pathway kinase MAP4K4. *Angew. Chem. Int. Ed. Engl.* 54 12398–12403. 10.1002/anie.201501515 25908259

[B52] Sood S. Gallagher I. J. Lunnon K. Rullman E. Keohane A. Crossland H. (2015). A novel multi-tissue RNA diagnostic of healthy ageing relates to cognitive health status. *Genome Biol.* 16:185. 10.1186/s13059-015-0750-x 26343147 PMC4561473

[B53] Stornetta R. L. Zhu J. J. (2011). Ras and Rap signaling in synaptic plasticity and mental disorders. *Neuroscientist* 17 54–78. 10.1177/1073858410365562 20431046 PMC3119507

[B54] Tan J. Xu Y. Han F. Ye X. (2019). Genetical modification on adipose-derived stem cells facilitates facial nerve regeneration. *Aging (Albany NY)* 11 908–920. 10.18632/aging.101790 30728320 PMC6382422

[B55] Tischer J. Krueger M. Mueller W. Staszewski O. Prinz M. Streit W. J. (2016). Inhomogeneous distribution of Iba-1 characterizes microglial pathology in Alzheimer’s disease. *Glia* 64 1562–1572. 10.1002/glia.23024 27404378

[B56] Tong L. Balazs R. Thornton P. L. Cotman C. W. (2004). Beta-amyloid peptide at sublethal concentrations downregulates brain-derived neurotrophic factor functions in cultured cortical neurons. *J. Neurosci.* 24 6799–6809. 10.1523/JNEUROSCI.5463-03.2004 15282285 PMC6729714

[B57] Wei Z. Chen X. C. Song Y. Pan X. D. Dai X. M. Zhang J. (2016). Amyloid beta protein aggravates neuronal senescence and cognitive deficits in 5XFAD mouse model of Alzheimer’s disease. *Chin. Med. J. (Engl.).* 129 1835–1844. 10.4103/0366-6999.186646 27453234 PMC4976573

[B58] Westenberger A. Balck A. Klein C. (2019). Primary familial brain calcifications: genetic and clinical update. *Curr. Opin. Neurol.* 32 571–578. 10.1097/WCO.0000000000000712 31157644

[B59] Wilms H. Rosenstiel P. Sievers J. Deuschl G. Zecca L. Lucius R. (2003). Activation of microglia by human neuromelanin is NF-kappaB dependent and involves p38 mitogen-activated protein kinase: implications for Parkinson’s disease. *FASEB J.* 17 500–502. 10.1096/fj.02-0314fje 12631585

[B60] Wu C. Watts M. E. Rubin L. L. (2019). MAP4K4 activation mediates motor neuron degeneration in amyotrophic lateral sclerosis. *Cell Rep.* 26 1143–1156.e5. 10.1016/j.celrep.2019.01.019 30699345

[B61] Xiao G. Zhang X. Zhang X. Chen Y. Xia Z. Cao H. (2021). Aging-related genes are potential prognostic biomarkers for patients with gliomas. *Aging (Albany NY)* 13 13239–13263. 10.18632/aging.203008 33946049 PMC8148480

[B62] Xu Q. Chen Y. (2021). An aging-related gene signature-based model for risk stratification and prognosis prediction in lung adenocarcinoma. *Front. Cell. Dev. Biol.* 9:685379. 10.3389/fcell.2021.685379 34277626 PMC8283194

[B63] Yang Y. Yuan L. Yang M. Du X. Qin L. Wang L. (2021). Aberrant methylation of aging-related genes in asthma. *Front. Mol. Biosci.* 8:655285. 10.3389/fmolb.2021.655285 34136532 PMC8203316

[B64] Yao Z. Zhou G. Wang X. S. Brown A. Diener K. Gan H. (1999). A novel human STE20-related protein kinase, HGK, that specifically activates the c-Jun N-terminal kinase signaling pathway. *J. Biol. Chem.* 274 2118–2125. 10.1074/jbc.274.4.2118 9890973

