# Supplementary figures and images for: Identification and Validation of Aging-Related Genes in Alzheimer’s Disease
Source: Front Neurosci. 2022 May 9;16:905722. doi: 10.3389/fnins.2022.905722 (PMC9124812; doi:10.3389/fnins.2022.905722)

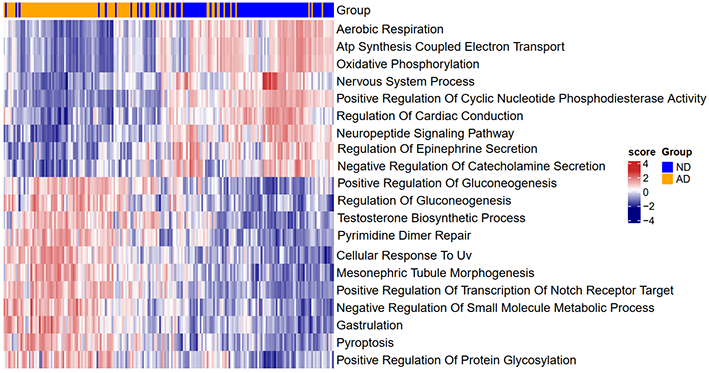

Supplement: Supplementary Figure 1 — Gene set variation analysis showed the 20 up and down regulated biological processes in AD patients. Regulation of gluconeogenesis, positive regulation of Notch receptor target, and pyroptosis processes were upregulated in AD, while nervous system process and neuropeptide signaling pathway were downregulated. [file Image_1.TIF]

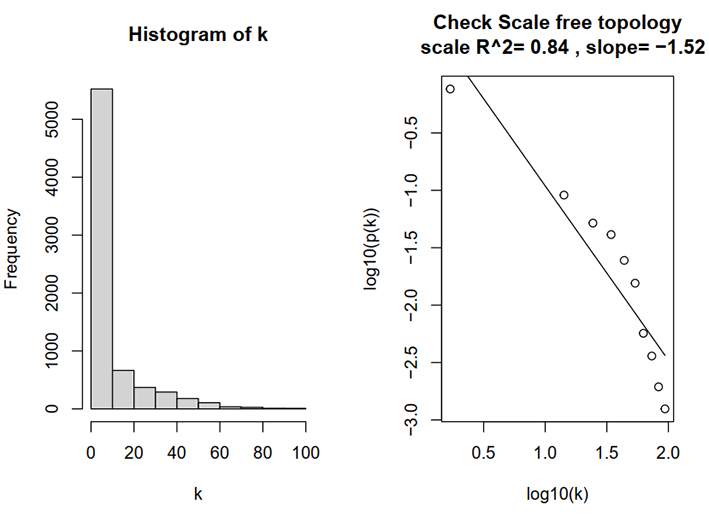

Supplement: Supplementary Figure 2 — Checking the scale-free topology using soft-thresholds with β = 22. [file Image_2.TIF]

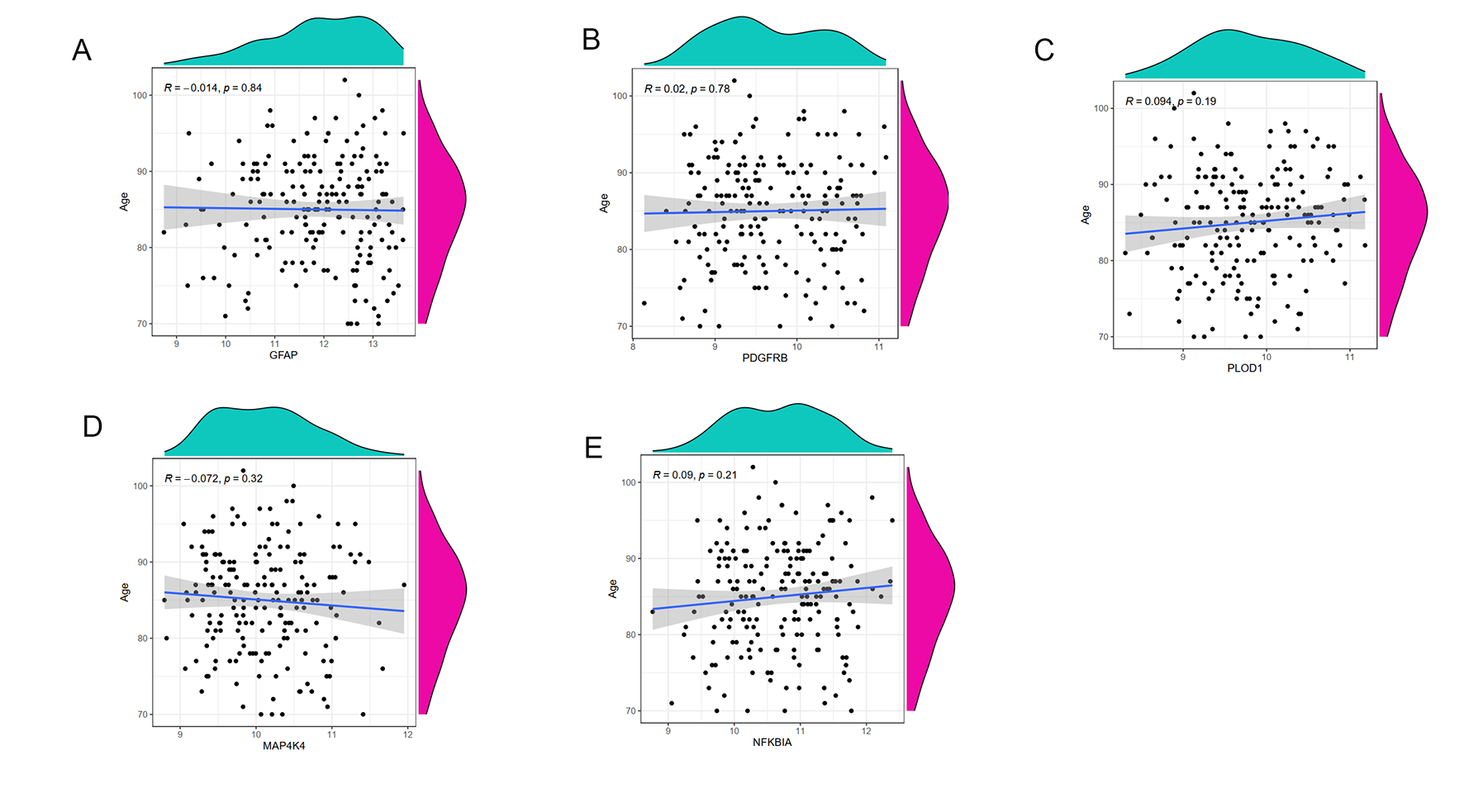

Supplement: Supplementary Figure 3 — Scatterplots showed no significant relationship between the five hub ARDEGs and age. Significance level was denoted by *p-value < 0.05, **p-value < 0.01, ***p-value < 0.001. [file Image_3.TIF]

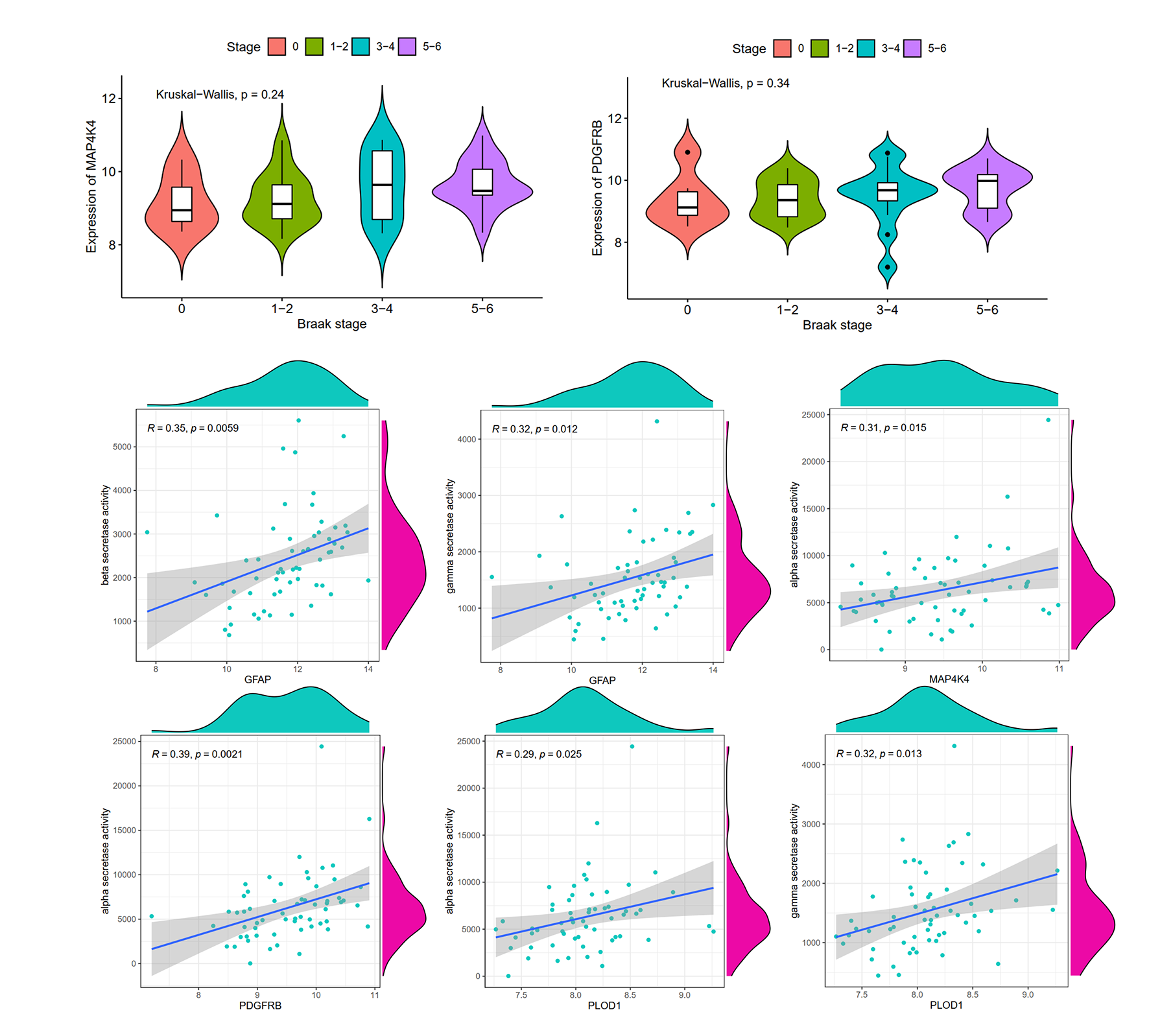

Supplement: Supplementary Figure 4 — (A,B) Showed the expression levels of MAP4K4 and PDGFRB in different Braak stages, respectively. (C–H) Scatterplots show the significant relationship between hub ARDEGs and clinical characteristics. Significance level was denoted by *p-value < 0.05, **p-value < 0.01, ***p-value < 0.001. [file Image_4.TIF]

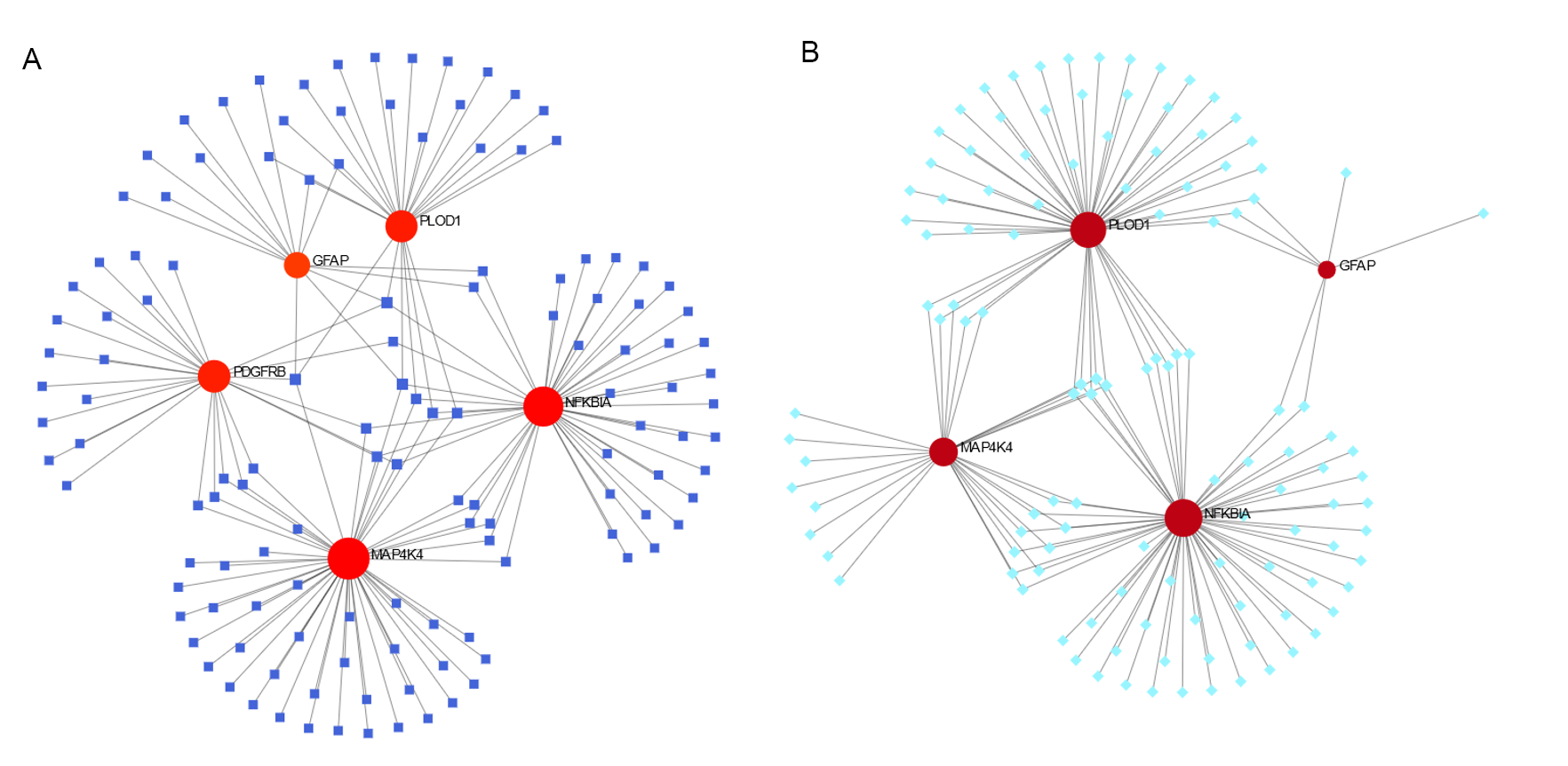

Supplement: Supplementary Figure 5 — The Predicted microRNA (A) and transcription factors (B) for the selected five hub ARDEGs. The circular nodes represent the ARDEGs, and the square nodes represent miRNA (A) or transcription factors (B), respectively. The size of a node depends on the degree of the node. [file Image_5.TIF]
