# Supplementary material for: Identification and Validation of Aging-Related Genes in Alzheimer’s Disease
Source: Front Neurosci. 2022 May 9;16:905722. doi: 10.3389/fnins.2022.905722 (PMC9124812; doi:10.3389/fnins.2022.905722)
Supplement: Supplementary file 6 [file Table_1.docx]

Table S1. Clinical characteristics of the samples

|  | AD (n=15) | Control (n=15) | *P* |
| --- | --- | --- | --- |
| Mean age (Mean±SD, year) | 63.53±9.76 | 63.33±6.25 | 0.95 |
| Sex (F/M, n) | 9/6 | 9/6 | 1.00 |
| Hypertension, % | 33.3% | 26.7% | 0.70 |
| Smoking, % | 20% | 20% | 1.00 |
| Excessive drinking, % | - | - | - |
| MMSE (Mean±SD) | 17.25±8.23 | - | - |
| MoCA (Mean±SD) 14.25±8.15 - - | | | |
| ADL (Mean±SD) | 27.85±11.7 | - | - |
| CDR (Mean±SD) | 0.81±0.7 | - | - |
