# Supplementary material for: Identification and Validation of Aging-Related Genes in Alzheimer’s Disease
Source: Front Neurosci. 2022 May 9;16:905722. doi: 10.3389/fnins.2022.905722 (PMC9124812; doi:10.3389/fnins.2022.905722)
Supplement: Supplementary file 7 [file Table_2.docx]

Table S2. The descriptions of aging-related differentially expressed genes in AD

| Gene Symbol | Co-efficient | Chromosome | Definition |
| --- | --- | --- | --- |
| MAP4K4 | 0.446 | 2 | Homo sapiens mitogen-activated protein kinase kinase kinase kinase 4 (MAP4K4), transcript variant 2, mRNA. |
| PLOD1 | 0.332 | 1 | Homo sapiens procollagen-lysine 1, 2-oxoglutarate 5-dioxygenase 1 (PLOD1), mRNA. |
| GFAP | 0.025 | 17 | Homo sapiens glial fibrillary acidic protein (GFAP), mRNA. |
| NFKBIA | 1.380 | 14 | Homo sapiens nuclear factor of kappa light polypeptide gene enhancer in B-cells inhibitor, alpha (NFKBIA), mRNA. |
| PDGFRB | 0.300 | 5 | Homo sapiens platelet-derived growth factor receptor, beta polypeptide (PDGFRB), mRNA. |
| GOT1 | - | 10 | Homo sapiens glutamic-oxaloacetic transaminase 1, soluble (aspartate aminotransferase 1) (GOT1), mRNA. |
| GABBR2 | - | 9 | Homo sapiens gamma-aminobutyric acid (GABA) B receptor, 2 (GABBR2), mRNA. |
| MLKL | - | 16 | Homo sapiens mixed lineage kinase domain-like (MLKL), mRNA. |
| YWHAZ | - | 8 | Homo sapiens tyrosine 3-monooxygenase/tryptophan 5-monooxygenase activation protein, zeta polypeptide (YWHAZ), transcript variant 1, mRNA. |
| TSPAN13 | - | 7 | Homo sapiens tetraspanin 13 (TSPAN13), mRNA. |
| DYNLT3 | - | X | Homo sapiens dynein, light chain, Tctex-type 3 (DYNLT3), mRNA. |
| MAP2K1 | - | 15 | Homo sapiens mitogen-activated protein kinase kinase 1 (MAP2K1), mRNA. |
| ITPKB | - | 1 | Homo sapiens inositol 1,4,5-trisphosphate 3-kinase B (ITPKB), mRNA. |
| SNCA | - | 4 | Homo sapiens synuclein, alpha (non A4 component of amyloid precursor) (SNCA), mRNA |
| Gene Symbol | Co-efficient | Chromosome | Definition |
| FOXO4 | - | X | Homo sapiens forkhead box O4 (FOXO4), mRNA. |
| OPA1 | - | 3 | Homo sapiens optic atrophy 1 (autosomal dominant) (OPA1), nuclear gene encoding mitochondrial protein, transcript variant 1, mRNA. |
| DCLK1 | - | 13 | Homo sapiens doublecortin-like kinase 1 (DCLK1), mRNA. |
| SLC16A14 | - | 2 | Homo sapiens solute carrier family 16, member 14 (monocarboxylic acid transporter 14) (SLC16A14), mRNA. |
| BCL6 | - | 3 | Homo sapiens B-cell CLL/lymphoma 6 (zinc finger protein 51) (BCL6), transcript variant 1, mRNA. |
| MXD4 | - | 4 | Homo sapiens MAX dimerization protein 4 (MXD4), mRNA. |
| UCHL1 | - | 4 | Homo sapiens ubiquitin carboxyl-terminal esterase L1 (ubiquitin thiolesterase) (UCHL1), mRNA. |
| SCOC | - | 4 | Homo sapiens short coiled-coil protein (SCOC), mRNA. |
| RAB13 | - | 1 | Homo sapiens RAB13, member RAS oncogene family (RAB13), mRNA. |
| ELMOD1 | - | 11 | Homo sapiens ELMO/CED-12 domain containing 1 (ELMOD1), mRNA. |
| SST | - | 3 | Homo sapiens somatostatin (SST), mRNA. |
| MAP2K4 | - | 17 | Homo sapiens mitogen-activated protein kinase kinase 4 (MAP2K4), mRNA. |
| GABARAPL1 | - | 12 | Homo sapiens GABA(A) receptor-associated protein like 1 |
