# Supplementary material for: Identification and Validation of Aging-Related Genes in Alzheimer’s Disease
Source: Front Neurosci. 2022 May 9;16:905722. doi: 10.3389/fnins.2022.905722 (PMC9124812; doi:10.3389/fnins.2022.905722)
Supplement: Supplementary file 8 [file Table_3.docx]

Table S3. The predicted upstream microRNA and transcription factor for the five aging-related differentially expressed genes.

| Gene | microRNA | Transcription factor |
| --- | --- | --- |
| NFKBIA | hsa-mir-24-3p,hsa-mir-196a-5p,hsa-mir-34a-5p,hsa-mir-23b-3p,hsa-mir-124-3p,hsa-mir-141-3p,hsa-mir-34c-5p,hsa-mir-335-5p,hsa-mir-122-5p,hsa-mir-18a-5p,hsa-mir-21-3p,hsa-mir-27a-3p,hsa-mir-27b-3p,hsa-mir-362-3p,hsa-mir-1-3p,hsa-mir-155-5p,hsa-mir-101-3p,hsa-mir-374a-5p,hsa-let-7g-3p,hsa-mir-128-3p,hsa-mir-129-1-3p,hsa-mir-129-2-3p,hsa-mir-130a-3p,hsa-mir-140-3p,hsa-mir-150-3p,hsa-mir-200a-3p,hsa-mir-29b-1-5p,hsa-mir-329-3p,hsa-mir-338-5p,hsa-mir-483-3p,hsa-mir-489-3p,hsa-mir-513a-5p,hsa-mir-548b-5p,hsa-mir-548c-5p,hsa-mir-548d-5p,hsa-mir-548f-3p,hsa-mir-548i,hsa-mir-548q,hsa-mir-548y,hsa-mir-7-1-3p,hsa-mir-940,hsa-mir-598-3p,hsa-mir-191-5p,hsa-mir-20a-5p,hsa-mir-212-3p,hsa-mir-27a-5p,hsa-mir-18b-5p | NR2F1,NFIA,RERE,ZFP64,HMG20A,CREM,ELF3,RARA,THRB,TEAD3,ZNF197,ZNF580,TEAD1,BCL6,FOSL1,ZNF7,KDM1A,SPI1,MXD3,HBP1,ATF3,NR3C1,STAT1,RCOR2,TCF7,GATA4,RUNX3,SSRP1,MAZ,TRIM22,DRAP1,KLF7,NFYC,MBD1,ZNF384,MXI1,HHEX,DMAP1,ETV4,BHLHE40,RELA,TFDP1,ZBTB26,TFAP4,MLX,GMEB2,RFX3,KLF8,HMG20B,SIN3A,KLF9,WRNIP1,SOX13,MXD4,ZNF644,ZNF423,ZHX2,CBFB,ZNF143,SUPT5H,ZNF207,ZKSCAN1,CEBPG,JUNB,KAT2A |
| PDGFRB | hsa-mir-29a-3p,hsa-mir-29b-3p,hsa-mir-34a-5p,hsa-mir-30b-5p,hsa-mir-9-5p,hsa-mir-29c-3p,hsa-mir-34c-5p,hsa-mir-767-5p,hsa-mir-145-5p,hsa-mir-16-5p,hsa-mir-27a-3p,hsa-mir-27b-3p,hsa-mir-101-3p,hsa-mir-30a-5p,hsa-mir-30c-5p,hsa-mir-30d-5p,hsa-mir-30e-5p,hsa-mir-337-3p,hsa-mir-449a,hsa-mir-449b-5p,hsa-mir-5581-3p,hsa-mir-744-5p,hsa-mir-376a-5p,hsa-mir-941,hsa-mir-34b-5p,hsa-mir-133a-3p | NA |
| PLOD1 | hsa-mir-192-5p,hsa-mir-34a-5p,hsa-mir-23b-3p,hsa-mir-124-3p,hsa-mir-484,hsa-mir-16-5p,hsa-mir-1-3p,hsa-mir-374a-5p,hsa-mir-103a-3p,hsa-mir-106a-5p,hsa-mir-107,hsa-mir-142-3p,hsa-mir-148b-5p,hsa-mir-3179,hsa-mir-339-3p,hsa-mir-3615,hsa-mir-423-3p,hsa-mir-522-5p,hsa-mir-576-3p,hsa-mir-7-5p,hsa-mir-146a-5p,hsa-mir-147a,hsa-mir-200c-3p,hsa-mir-214-3p,hsa-mir-671-5p | ZBTB33,GFI1B,RXRB,CREM,ELF3,THRB,ELF1,ZNF580,IRF1,NFRKB,CREB1,KLF6,ZNF7,GATA4,NFYC,RELA,TFDP1,MLX,E2F5,TAL1,KLF16,NCOA1,BCL11B,KLF11,BCL11A,SP1,ARNT,PRDM1,CUX1,HMGN3,WT1,ZNF501,ESRRA,ZNF24,EGR1,HES1,MNT,BCOR,HDGF,MIXL1,DDX20,NR4A1,SMARCA5,USF1,USF2,TAF7,BACH1,MEF2D,RAD51,ZNF394,IRF4,HCFC1,ELK1,FOXM1,NCOR1,ZEB1 |

Continued

| MAP4K4 | hsa-mir-29a-3p,hsa-mir-29b-3p,hsa-mir-181a-5p,hsa-mir-23b-3p,hsa-mir-124-3p,hsa-mir-141-3p,hsa-mir-9-5p,hsa-mir-29c-3p,hsa-mir-122-5p,hsa-mir-1226-3p,hsa-mir-1249-3p,hsa-mir-1299,hsa-mir-141-5p,hsa-mir-145-5p,hsa-mir-149-3p,hsa-mir-16-5p,hsa-mir-181a-2-3p,hsa-mir-18a-5p,hsa-mir-1913,hsa-mir-192-3p,hsa-mir-21-3p,hsa-mir-24-2-5p,hsa-mir-27a-3p,hsa-mir-27b-3p,hsa-mir-30a-3p,hsa-mir-30e-3p,hsa-mir-323a-3p,hsa-mir-346,hsa-mir-362-3p,hsa-mir-3657,hsa-mir-3928-3p,hsa-mir-452-5p,hsa-mir-490-3p,hsa-mir-516b-5p,hsa-mir-518c-5p,hsa-mir-548at-5p,hsa-mir-548b-3p,hsa-mir-550a-3p,hsa-mir-592,hsa-mir-597-5p,hsa-mir-622,hsa-mir-766-5p,hsa-mir-873-5p,hsa-mir-877-3p,hsa-mir-93-3p,hsa-mir-135a-5p,hsa-mir-23a-3p,hsa-mir-26a-5p,hsa-mir-1-3p,hsa-mir-155-5p,hsa-mir-101-3p,hsa-mir-210-3p,hsa-mir-429,hsa-mir-374a-5p | RERE,ZFP64,HMG20A,NFIL3,CREM,GATAD2A,ELF3,TGIF2,RARA,CEBPB,THRB,TEAD3,ELF1,ZNF197,YY1,TFE3,CTCF,ZNF580,TEAD1,IRF1,BCL6,NFRKB,CREB1,FOSL1,KLF6,L3MBTL2,ZNF7,KDM1A |
| --- | --- | --- |
| GFAP | hsa-mir-24-3p,hsa-mir-34a-5p,hsa-mir-124-3p,hsa-mir-335-5p,hsa-mir-16-5p,hsa-mir-103a-3p,hsa-mir-107,hsa-mir-15a-5p,hsa-mir-15b-5p,hsa-mir-195-5p,hsa-mir-324-5p,hsa-mir-338-3p,hsa-mir-497-5p,hsa-mir-1343-3p | NR2F1,NFIA,ZBTB33,GATAD1,FOSL2,GFI1B |
